# Nuclear Ep-ICD accumulation predicts aggressive clinical course in early stage breast cancer patients

**DOI:** 10.1186/1471-2407-14-726

**Published:** 2014-09-29

**Authors:** Gunjan Srivastava, Jasmeet Assi, Lawrence Kashat, Ajay Matta, Martin Chang, Paul G Walfish, Ranju Ralhan

**Affiliations:** Alex and Simona Shnaider Research Laboratory in Molecular Oncology, Mount Sinai Hospital, 600 University Avenue, Suite 6-318, Toronto, M5G 1X5 Ontario Canada; Department of Pathology and Laboratory Medicine, Mount Sinai Hospital, Toronto, M5G 1X5 Ontario Canada; Department of Laboratory Medicine and Pathobiology, University of Toronto, Toronto, M5S 1A8 ON Canada; Joseph and Mildred Sonshine Family Centre for Head and Neck Diseases, Mount Sinai Hospital, Toronto, Ontario Canada; Department of Medicine, Endocrine Division, Mount Sinai Hospital and University of Toronto, Toronto, M5G 1X5 Ontario Canada; Department of Otolaryngology-Head and Neck Surgery, Mount Sinai Hospital, Toronto, Ontario Canada; Joseph and Mildred Sonshine Family Centre for Head and Neck Diseases, Department of Otolaryngology-Head and Neck Surgery Program, Room 413, Joseph & Wolf Lebovic Health Complex, 600 University Avenue, Mount Sinai Hospital, Toronto, M5G 1X5 Ontario Canada

**Keywords:** Breast cancer, Ductal carcinoma in situ, Invasive ductal carcinoma, Invasive lobular carcinoma, Invasive mucinous carcinoma, Lobular carcinoma in situ, EpCAM, Ep-ICD and EpEx

## Abstract

**Background:**

Regulated intramembrane proteolysis of Epithelial cell adhesion molecule (EpCAM) results in release of its intracellular domain (Ep-ICD) which triggers oncogenic signalling. The clinical significance of Ep-ICD in breast cancer remains to be determined. Herein, we examined the expression of nuclear and cytoplasmic Ep-ICD, and membranous extracellular domain of EpCAM (EpEx) in breast cancer patients, to determine its potential utility in predicting aggressive clinical course of the disease.

**Methods:**

In this retrospective study, 266 breast cancers and 45 normal breast tissues were immunohistochemically analyzed to determine the expression patterns of nuclear and cytoplasmic Ep-ICD and membranous EpEx and correlated with clinicopathological parameters and follow up. Disease-free survival was determined by Kaplan-Meier method and multivariate Cox regression analysis.

**Results:**

Nuclear Ep-ICD was more frequently expressed in breast cancers compared to normal tissues. Significant association was observed between increased nuclear Ep-ICD expression and reduced disease-free survival in patients with ductal carcinoma in situ (DCIS) and invasive ductal carcinoma (IDC) (p < 0.001). Nuclear Ep-ICD was positive in all the 13 DCIS and 25 IDC patients who had reduced disease-free survival, while none of the nuclear Ep-ICD negative DCIS or IDC patients had recurrence during the follow up period. Notably, majority of IDC patients who had recurrence had early stage tumors. Multivariate Cox regression analysis identified nuclear Ep-ICD as the most significant predictive factor for reduced disease-free survival in IDC patients (p = 0.011, Hazard ratio = 80.18).

**Conclusion:**

Patients with nuclear Ep-ICD positive breast cancers had poor prognosis. The high recurrence of disease in nuclear Ep-ICD positive patients, especially those with early tumor stage suggests that nuclear Ep-ICD accumulation holds the promise of identifying early stage patients with aggressive disease who are likely to be in need of more rigorous post-operative surveillance and/or treatment.

## Background

Breast cancer is the most frequently diagnosed cancer in females, with an estimated 1.38 million new cases per year worldwide [1, 2] and an estimated 226 870 new cases in the United States in 2012 [1, 2]. Globally, there are 458 000 deaths per year from this malignancy making it the most common cause of cancer death in women in both the developed and developing countries [1]. In early stage breast carcinoma patients, the presence of metastases to axillary lymph nodes is the most important predictor of survival [3]. Patients with node-positive tumors have up to an 8-fold increase in mortality than node-negative patients [4]. The heterogenic nature of breast carcinomas and diverse patterns of growth and invasiveness emphasize the need for prognostic and predictive biological markers for aggressive tumors. This is particularly important in light of the fact that many detected carcinomas may be non-aggressive [5]. Furthermore, population breast cancer screening with mammography may facilitate early detection of breast tumors and has the potential to lower mortality, but it is also associated with the risk of overtreatment of less aggressive subtypes resulting in unnecessary treatment of tumors that would not have adversely affected the patient [6]. Therefore, it is important to identify more aggressive lesions at an earlier stage for rigorous treatment.

Current clinical therapies for breast cancer include surgery, radiotherapy and drug therapies targeting oncogenic processes that are offered on an individual patient basis. The prediction of treatment response and propensity for metastasis remain challenging, and reflect an incomplete understanding of the biology of different breast cancer subtypes. A large number of patients are over-treated to achieve improved overall survival in early breast cancer. Defining individual risk of disease recurrence or sensitivity to treatment will considerably reduce over-treatment and enable personalised treatment so that patients only receive the optimal treatment required to achieve the cure. Genomic tests (Mammaprint, Oncotype Dx, PAM50) and immunohistochemical tests (IHC 4) have been developed for prediction of disease prognosis and response to chemotherapy; prospective validation of these is still awaited [7]. Nuclear magnetic resonance (NMR) and mass spectrometry (MS) based serum metabolite profiling has been shown to accurately identify 80% of breast cancer patients whose tumors failed to respond to chemotherapy suggesting promise for personalised treatment protocols [8]. Recently, a five-gene Integrated Cytokine score (ICS) has been proposed for predicting metastatic outcome from primary hormone receptor negative and/or triple negative breast tumors independent of nodal status, adjuvant chemotherapy use, and triple negative molecular subtype [9].

Epithelial Cell Adhesion Molecule (EpCAM) is a transmembrane glycoprotein expressed in several human epithelial tissues and frequently overexpressed in cancer, progenitor, and stem cells [10]. EpCAM consists of an extracellular epidermal growth factor-like (EGF) domain (EpEx), thyroglobulin domain, transmembrane region, and a short intracellular domain (Ep-ICD) [11, 12]. In normal cells, EpCAM appears to be sequestered in tight junctions and is therefore less accessible to antibodies, whereas in cancer cells it is widely distributed on the cell surface and has therefore been explored as a surface-binding site for therapeutic antibodies [13–16]. EpCAM has been widely investigated for its diagnostic and therapeutic potential as it is expressed in the majority of human epithelial cancers, including breast, colon, gastric, head and neck, prostate, pancreas, ovarian and lung cancer [17–20]. Increased EpCAM expression has been found to be a poor prognostic marker in breast and gall bladder carcinomas [21, 22]. In contrast EpCAM expression in colorectal and gastric cancer is associated with favorable prognosis [23, 24]. This paradoxical association of EpCAM expression with prognosis in different cancers is supported by functional studies of EpCAM biology using in vitro and in vivo cancer models as well. Taken together these studies suggest that the impact of EpCAM expression in human cancers is likely to be context dependent [25]. EpCAM expression based assay has been FDA approved and widely used to detect circulating tumor cells in breast cancer [26]. Due to its high-expression and association with poor prognosis, EpCAM has been widely explored as a potential target for antibody-based immunotherapies. EpCAM-targeted molecular therapies are being intensely pursued for several cancers including breast, ovarian, gastric and lung cancer [27]. EpCAM expression has been used to predict response to anti-EpCAM antibodies in breast cancer patients [27–29]. Surprisingly clinical trials of anti-EpCAM antibodies targeting the EpEx domain have shown limited efficacy [29, 30]. These paradoxical outcomes are potentially explainable by the recently described regulated intramembrane proteolysis of EpCAM, resulting in oncogenic signaling by its intracellular domain, Ep-ICD [31]. Previously, we reported accumulation of Ep-ICD is frequently detected in ten epithelial cancers, including breast and prostate [32, 33]. In thyroid carcinomas nuclear Ep-ICD accumulation predicted poor prognosis and was elevated in patients with anaplastic tumors [33].

The aim of this study is to evaluate the prognostic utility of Ep-ICD by characterizing the subcellular expression of Ep-ICD and EpEx in breast carcinomas using immunohistochemistry and correlating with clinicopathological parameters and the follow up of patients to investigate its potential to predict aggressive tumors that may aid in the management of breast cancer patients.

## Methods

### Patient and tumor specimens

This retrospective study of biomarkers using the breast cancer patients’ tissue blocks stored in the archives of the Department of Pathology and Laboratory Medicine and their anonymized clinical data was approved by the Mount Sinai Hospital Research Ethics Board, Toronto, Canada. The patients whose records were used for this study granted informed consent for their tissue samples to be archived and used for research purposes. In view of the retrospective study, the need for consent for use of anonymized clinical data was waived-off by the Institutional Research Ethics Board. The patient cohort consisted of 266 breast cancer patients treated at Mount Sinai Hospital, a tertiary care hospital in Toronto, Ontario, Canada between 2000 and 2007. The series consisted of patients who had mastectomy or lumpectomy. Inclusion criteria: Breast cancer tissue samples of patients that had up to 60 months follow-up and availability of clinical, pathological and treatment data in the clinical database.

Exclusion criteria: Breast cancer tissues were not considered for this study if patients follow up data were not available in the clinical database.

Normal breast tissues were chosen from breast reduction surgeries, normal tissue with adjacent benign lesions, and prophylactic mastectomies. Normal breast tissues from adjacent cancers were not included in this study. Our patient cohort consisted of individuals with invasive ductal carcinoma (IDC) (n = 180), invasive lobular carcinoma (ILC) (n = 15), invasive mucinous carcinoma (IMC) (n = 9), ductal carcinoma in situ (DCIS) (n = 61), and lobular carcinoma in situ (LCIS) (n = 1) and 45 individuals with normal breast tissues. The diagnosis was based on histopathological analysis of the tissue specimens. The follow-up time for all patients including IDC cases in the study was 60 months. The clinicopathological parameters recorded included age at surgery, tumor histotype, tumor size, AJCC pTNM stage, nodal status, tumor grade, recurrence of disease, ER/PR status, hormonal treatment, radiation therapy, and/or chemotherapy. Her2 status data were not available for all breast cancer patients in the clinical database and thus could not be included in this study. Formalin-fixed paraffin-embedded tissue blocks of all patients included in this study were retrieved from the Mount Sinai Hospital (MSH) tumor bank, reviewed by the pathologists and used for cutting tissue sections for immunohistochemical staining with Ep-ICD and EpEx specific antibodies as described below.

### Immunohistochemistry (IHC)

Formalin-fixed paraffin embedded sections (4 μm thickness) of breast carcinomas were used for Ep-ICD and EpEx immunostaining as described [33]. In brief, for EpEx following deparaffinization and rehydration, antigen retrieval was carried out using a microwave oven in 0.01 M citrate buffer, pH 3.0 and endogenous peroxidase activity was blocked by incubating the tissue sections in hydrogen peroxide (0.3%, v/v) for 20 min. For Ep-ICD, the tissue sections were de-paraffinized by baking at 62°C for 1 hour in vertical orientation, treated with xylene and graded alcohol series, and the non-specific binding was blocked with normal horse or goat serum. Rabbit anti-human Ep-ICD monoclonal antibody from Epitomics Inc. (Burlingame, CA) was used in this study. The α-Ep-ICD antibody 1144 recognizes the cytoplasmic domain of human EpCAM and has been used in our previous study of Ep-ICD expression in thyroid carcinoma and other epithelial cancers [33]. Anti-EpCAM monoclonal antibody EpEx (MOC-31, AbD Serotec, Oxford, UK) recognizes an extracellular component (EGF1 domain- aa 27–59) in the amino-terminal region [34]. The sections were incubated with either α-Ep-ICD rabbit monoclonal antibody 1144 (dilution 1:1500) or mouse monoclonal antibody MOC-31 (dilution 1:200) for 60 minutes, followed by biotinylated secondary antibody (goat anti-rabbit or goat anti-mouse) for 20 minutes. The sections were finally incubated with VECTASTAIN Elite ABC Reagent (Vector Laboratories, Burlington, ON, Canada) and diaminobenzidine was used as the chromogen. Tissue sections were then counterstained with hematoxylin. Negative controls comprised of breast tissue sections incubated with isotype specific IgG in place of the primary antibody, and positive controls (colon cancer tissue sections known to express Ep-ICD) were included with each batch of staining for both Ep-ICD and EpEx.

### Evaluation of IHC and scoring

Immunopositive staining was evaluated in five areas of the tissue sections representing the highest tumor grade (Nottingham system) by two researchers blinded to the final outcome and the average of these five scores was calculated as described by us [33]. Sections were scored on the basis of both the percentage of immunopositive cells and intensity of staining. For percentage positivity, cells were assigned scores based on the following scheme: 0, < 10% cells; 1, 10–30% cells; 2, 31–50% cells; 3, 51–70% cells; and 4, >70% cells showing immunoreactivity. Sections were also scored semi-quantitatively on the basis of intensity of staining as follows: 0, none; 1, mild; 2, moderate; and 3, intense. A final score (ranging from 0 to 7) for each tissue section was obtained by adding the scores of percentage positivity and intensity for each of the breast cancer tissue sections. The average total score from the five areas was used for further statistical analysis. Each tissue section was scored for cytoplasmic and nuclear Ep-ICD as well as for membrane EpEx following this scoring scheme.

### Statistical analysis

The immunohistochemical data were subjected to statistical analysis with SPSS 21.0 software (SPSS, Chicago, IL) and GraphPad Prism 6.02 software (GraphPad Software, La Jolla, CA) as described previously [35]. A two-tailed p-value was used in all analyses and a p value < 0.05 was considered statistically significant. Chi-square analysis was used to determine the relationship between Ep-ICD and EpEx expression and the clinicopathological parameters. Disease-free survival was analyzed by the Kaplan-Meier method and multivariate Cox regression. Hazard ratios (HR), 95% confidence intervals (95% CI), and p values were estimated using the log-rank test. Disease-free survival or clinical recurrence, distal metastases, and/or death were considered to be the endpoint of the study. The cut-offs for statistical analysis were based upon the optimal sensitivity and specificity obtained from the Receiver operating curves as described [32]. For nuclear Ep-ICD, an IHC score cut-off value of ≥ 2 was defined as immunopositive for all tissues analyzed for statistical analysis. Ep-ICD cytoplasmic positivity was considered positive with an IHC cut-off value of ≥ 4. Membranous EpEx positivity was defined as membrane EpEx IHC score of ≥ 3.

## Results

The clinicopathological parameters and treatment details of all the 266 breast cancer patients and 45 normal controls are summarized in Table 1. The median age of patients was 59.9 years (range 30.6–89.8 years). AJCC pTNM Stage I (35.3%) and II (32.7%) comprised a large proportion of tumors in this cohort. Tumor grades distribution was Grade I - 21.1%; II - 39.8%, and III - 32.0%. Among the IDC cases, majority were also AJCC pTNM Stage I (62.8%) and II (32.2%). The IDC cases comprised of Grade I - 23.3%; Grade II - 36.7%; and Grade III - 36.1% tumors.

**Table 1:** **Clinicopathological characteristics of breast cancer patients in the study cohort**

	Breast cancer (n = 266)	IDC (n = 180)
**Surgical treatment**		
Lumpectomy	168 (63.1%)	113 (62.8%)
Mastectomy	84 (31.6%)	59 (32.8%)
Unknown	14 (5.3%)	8 (4.4%)
**Age at diagnosis (years)**		
Median (Range - 30.6–89.8)	59.2	59.2
< 59	126 (47.4%)	88 (48.9%)
≥ 59	140 (52.6)	92 (51.1%)
**Adjuvant treatment**		
Hormonal treatment		
Tamoxifen	131 (49.2%)	94 (52.2%)
Aromatase Inhibitor	13 (4.9%)	8 (4.4%)
Chemotherapy	73 (2.7%)	66 (24.8%)
Radiotherapy	149 (56.0%)	101 (56.1%)
Therapy details not available	6 (2.2%)	6 (3.3%)
**Tumor size (cm)**		
Mean ± SD	1.85 ± 1.525	1.82 ± 1.466
Minimum	0.1	0.1
Maximum	9	9
≤2 cm	198	81
>2 cm	57	96
Unknown	11	3
**AJCC pTNM stage (n, %)**		
0 (DCIS + LCIS)	62 (23.3%)	-
I	94 (35.3%)	113(62.8%)
II	87 (32.7%)	58 (32.2%)
III	6 (2.3%)	5 (2.8%)
IV	17 (6.4%)	4 (2.2%)
**Estrogen receptor (ER)**		
Negative	35 (13.1%)	33 (18.3%)
Positive	161 (60.6%)	136 (75.6%)
Unknown	70 (26.3%)	11 (6.1%)
**Progesterone receptor (PR)**		
Negative	71(26.7%)	64 (35.6%)
Positive	123 (46.2%)	103 (57.2%)
Unknown	72 (27.1%)	13 (7.2%)
**Grade**		
I	56 (21.1%)	42 (23.3%)
II	106 (39.8%)	66 (36.7%)
III	85 (32.0%)	65 (36.1%)
Unknown	19 (7.1%)	7 (3.9%)
**Nodal status**		
Negative	204 (76.7%)	123 (68.3%)
Positive	62 (23.3%)	57 (31.7%)

### Expression of Ep-ICD and EpEx in breast cancer tissues

**Figure 1:**
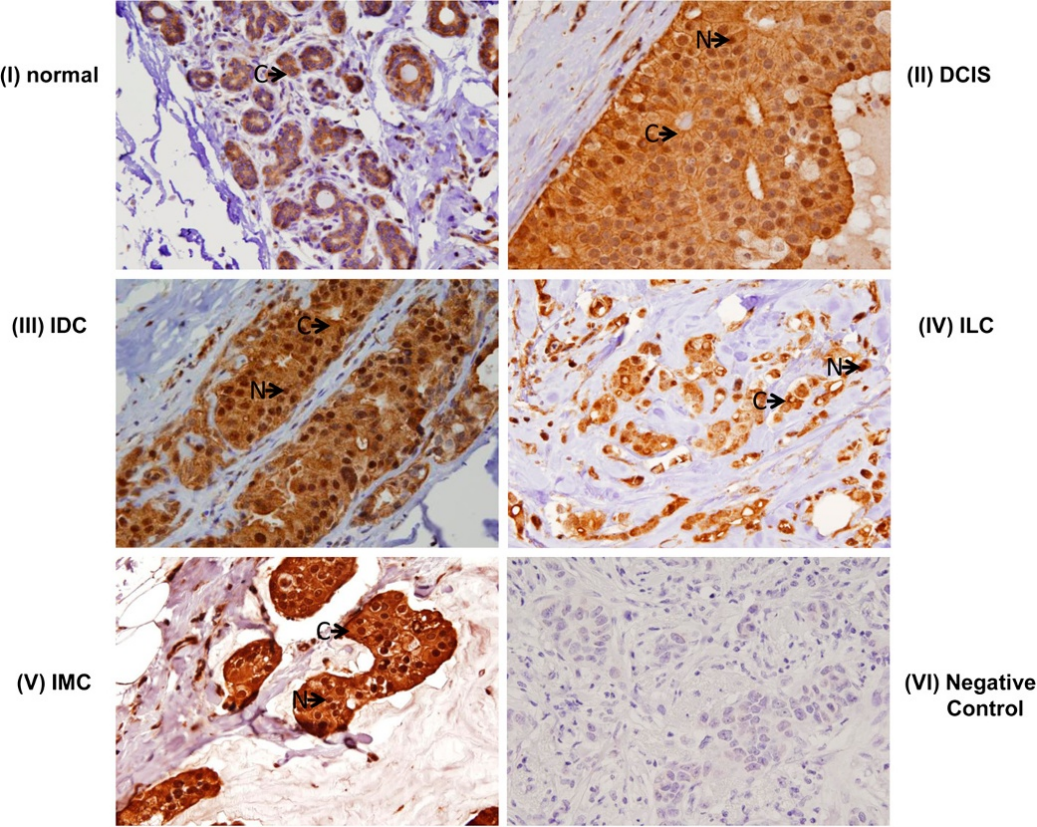
**Immunohistochemical analysis of Ep-ICD expression in breast cancer.** Representative photomicrographs demonstrating: **(I)** predominantly cytoplasmic Ep-ICD expression in normal breast tissues. Nuclear and cytoplasmic accumulation of Ep-ICD in: **(II)** DCIS; **(III)** IDC; **(IV)** ILC; **(V)** IMC; and **(VI)** negative control breast cancer tissue incubated with isotype specific IgG showing no detectable immunostaining for Ep-ICD. The arrows labelled N and C depict nuclear, and cytoplasmic staining respectively. (original magnification × 400).

**Figure 2:**
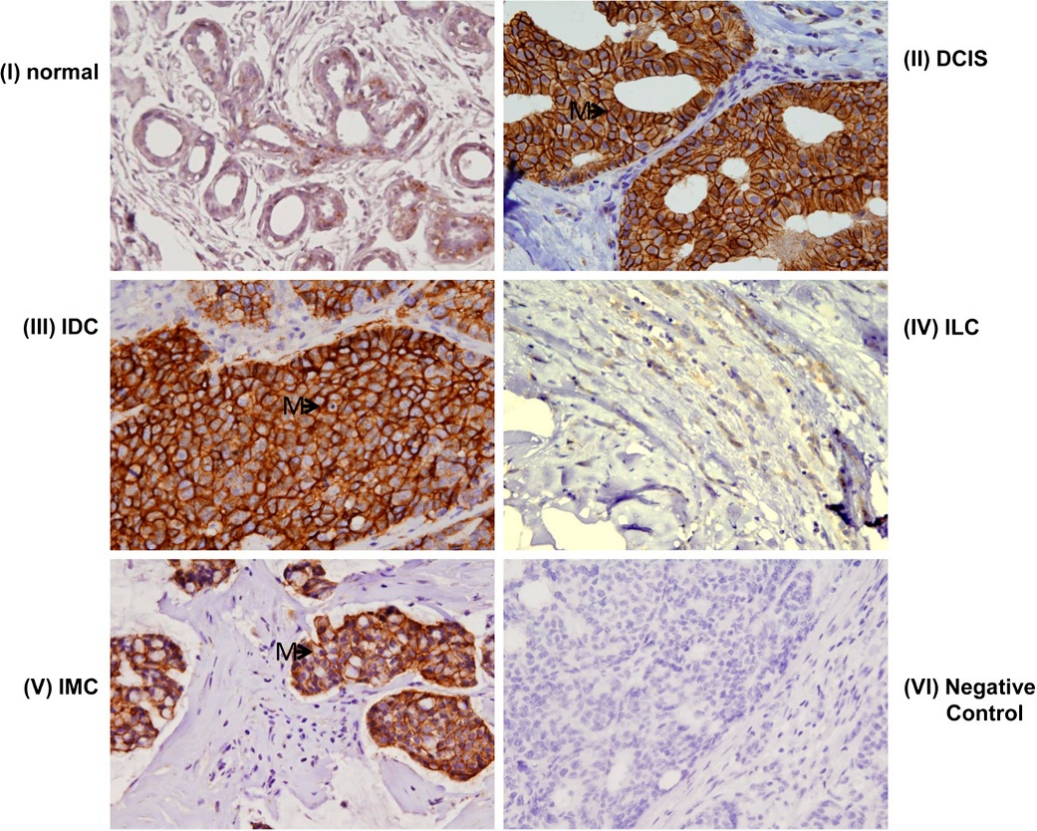
**Immunohistochemical analysis of EpEx expression in breast cancer.** Expression of EpEx in **(I)** normal breast tissues; **(II)** DCIS; **(III)** IDC; **(IV)** ILC; **(V)** IMC; (VI) negative control breast cancer tissue incubated with isotype specific IgG showing no detectable immunostaining for EpEX. Membranous EpEx expression was more frequently observed in breast carcinomas compared to normal tissues, except ILC (original magnification × 400). The arrows labelled M depict membrane staining.

To determine the pattern of expression of Ep-ICD and EpEx in breast cancer, tissues of DCIS, IDC, ILC, and IMC were analyzed by IHC and compared to normal breast tissues. A summary of the percentage positivity for nuclear Ep-ICD, cytoplasmic Ep-ICD, and membranous EpEx and loss of membranous EpEx is provided in Table 2. Representative photomicrographs of Ep-ICD and EpEx expression in breast cancer subtypes are shown in Figures 1 and 2. Of 266 breast carcinomas examined, 121 (46%) were positive for nuclear Ep-ICD and 185 (70%) were positive for membranous EpEx, while 81 cases showed loss of membranous EpEx expression. This compares to 11 of 45 (24%) normal breast tissues immunopositive for nuclear Ep-ICD and 19 of 45 (42%) positive for membranous EpEx. Notably, 12 of 15 ILCs showed loss of membranous EpEx, compared to 14 of 61 (23%) DCIS, 52 of 180 (29%) IDC and 3 of 9 IMC. Cytoplasmic Ep-ICD was frequently present in all histologic subtypes examined and normal tissues. Nuclear Ep-ICD was more frequently positive in breast carcinomas (121 of 266, 46%) compared to normal tissues (11 of 45, 24%). Evaluation of the individual subtypes showed nuclear Ep-ICD accumulation was frequently detected in ILC (10 of 15 tumors), 30 of 61 DCIS, 75 of 180 IDC, and 5 of 9 IMC cases.

**Table 2:** **Expression of nuclear and cytoplasmic Ep-ICD and membranous EpEx in normal tissues and breast cancer histotypes**

Tissue type	Number of tissues N	Nuclear Ep-ICD positivity n (%)	Cytoplasmic Ep-ICD positivity n (%)	Membranous EpEx positivity n (%)	Loss of membranous EpEx n (%)
**Normal**	45	11 (24% )	39 (87%)	19 (42%)	26 (58%)
**Breast cancer**	266	121 (46%)	215 (81%)	185 (70%)	81 (30%)
***Histotypes*** *					
**DCIS**	61 (22.9%)	30 (49%)	48 (79%)	47 (77%)	14 (23%)
**IDC**	180 (67.6%)	75 (42%)	145 (81%)	128 (71%)	52 (29%)
**ILC**	15	10	12	3	12
**IMC**	9	5	9	6	3

For nuclear Ep-ICD a cut off of ≥ 2 was used to determine positivity. For cytoplasmic Ep-ICD the cut off was ≥ 4. For membranous EpEx a cut off of ≥ 3 was considered positive.

*1 LCIS was also included in the study (data not shown in table).

### Relationship of Ep-ICD with clinicopathological characteristics of IDC patients

Nuclear and cytoplasmic Ep-ICD expression in IDC patients’ and their association with the clinicopathological characteristics are given in Table 3. Notably, nuclear Ep-ICD accumulation was significantly associated with and observed in all IDC patients with clinical recurrences [25 of 25 patients, p < 0.001, Odds ratio (OR) = 1.50, 95% confidence interval (CI) = 1.28–1.76]. Nuclear Ep-ICD overexpression was significantly associated with low or intermediate tumor grade (Grade I and II) (53 of 108 patients, 49%; p = 0.018, OR = 0.46, 95% CI = 0.24–0.89) and no lymph node metastases at surgery (58 of 123 patients, 47%; p = 0.028, OR = 0.48, 95% CI = 0.24–0.98). No association was observed between nuclear or cytoplasmic Ep-ICD and ER/PR status, AJCC pTNM stage, T-stage, tumor size, or patient’s age at diagnosis (Table 3). Membranous EpEx or loss of membranous EpEx did not show significant correlation with any of the clinico-pathological parameters in this cohort of breast cancer patients (data not shown).

**Table 3:** **Nuclear and cytoplasmic Ep-ICD expression in invasive ductal carcinoma (IDC) and correlation with clinicopathological parameters**

Clinicopathological parameters	Total cases (n = 180)	Ep-ICD Nuclear		p-value	Odd’s ratio (95% C.I.)	Ep-ICD Cytoplasm		p-value	Odd’s ratio (95% C.I.)
		N	(%)			n	(%)		
**IDC cases**		75	42	-	-	145	81	-	-
**Age**									
**< 59 years**	88	39	44.3			74	84.1		
**≥ 59 years**	92	36	39.1	0.480	0.80 (0.45–1.45)	71	77.2	0.241	0.64(0.30–1.36)
**Tumor Size** ^**a**^									
**≤2 cm**	81	35	43.2			69	85.2		
**> 2 cm**	96	37	38.5	0.529	0.82 (0.45–1.50)	73	76.0	0.128	0.55(0.25–1.20)
**T-stage**									
**T**_**1**_ **+ T**_**2**_	171	71	41.5			138	80.7		
**T**_**3**_ **+ T**_**4**_	9	4	44.4	0.862	1.13 (0.30–4.34)	7	77.8	0.829	0.84(0.17–4.22)
**Nodal Status**									
**N**_**x+0**_	123	58	47.2			99	80.5		
**N**_**1–3**_	57	17	29.8	**0.028**	0.48 (0.24–0.98)	46	80.7	0.973	1.02(0.45–2.24)
**Stage**									
**I + II**	159	68	42.8			130	81.8		
**III + IV**	21	7	33.3	0.410	0.67 (0.26–1.74)	15	71.4	0.261	0.56(0.20–1.56)
**Grade** ^**b**^									
**I + II**	108	53	49.1			90	83.3		
**III**	65	20	30.8	**0.018**	0.46 (0.24–0.89)	48	73.8	0.132	0.57(0.27–1.20)
**Clinical Recurrence**									
**No**	155	50	32.3			121	78.1		
**Yes**	25	25	100	**<0.001**	1.50 (1.28–1.76)	24	96.0	**0.035**	6.75(0.88–51.67)
**ER/ PR status** ^**c**^									
**ER** ^**+**^	136	62	45.6			112	82.4		
**ER** ^**-**^	33	12	36.4	0.338	1.47 (0.67–3.22)	25	75.8	0.386	1.49(0.60–3.71)
**PR** ^**+**^	103	49	47.6			88	85.4		
**PR** ^**-**^	64	25	39.1	0.282	1.42 (0.75–2.67)	48	75.0	0.092	1.96(0.89–4.30)
**ER** ^**+**^ **PR** ^**+**^	103	49	47.6			88	85.4		
**ER** ^**-**^ **PR** ^**-**^	33	12	36.4	0.260	1.59(0.70–3.56)	25	75.8	0.197	1.96(0.89–4.30)

^a^Tumor Size was available for 177 IDCs; ^b^Tumor Grades were available for 173 IDCs; ^c^ER and PR status was available for 169 and 167 IDCs only in our clinical databases. Membranous EpEx expression or loss of Membranous EpEx did not show significant correlation with any clinical or pathological parameters, hence the data are not shown in this Table. The p-value in boldface are statically significant.

It is important to note that recurrence, distal metastases, and/or death was observed in 42 of 121 (34.7%) breast carcinoma patients. Subgroup analysis of IDC patients that were positive for nuclear Ep-ICD showed recurrence in 25 of 75 (33.3%) patients. Importantly, in the entire cohort of breast carcinoma patients, only patients who were positive for nuclear Ep-ICD accumulation had disease recurrence. Notably, evaluation of all patients who had recurrence showed that of these 42 patients, 37 (88.1%) had early stage tumors (AJCC pTNM Stage I or II), while 5 (11.9%) were Stage III or IV tumors. Among the 25 IDC patients who had adverse clinical events, 21 of 25 (84%) had early stage tumors (AJCC pTNM Stage I and II), while 4 of 25 (16%) were AJCC pTNM Stage III and IV cases.

### Prognostic value of Ep-ICD expression for disease-free survival

**Figure 3:**
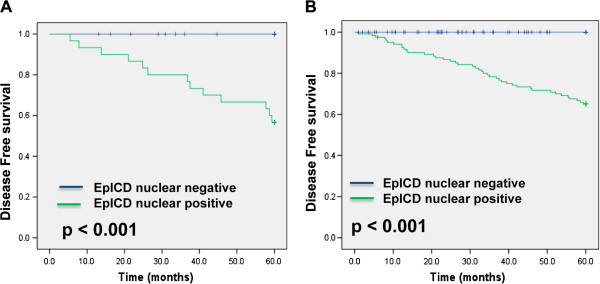
**Kaplan-Meier curves for disease-free survival (DFS) stratified by nuclear Ep-ICD expression in DCIS and in IDC. A**. Nuclear accumulation of Ep-ICD was associated with significantly reduced DFS in DCIS patients (p < 0.001). In 30 patients positive for nuclear Ep-ICD accumulation, 13 recurrences of DCIS were observed. In contrast, no recurrence was observed in 31 patients who did not show nuclear Ep-ICD immunopositivity and these patients were recurrence free for 60 months. **B**. Nuclear accumulation of Ep-ICD was associated with significantly reduced DFS in IDC patients (p < 0.001). In 75 patients positive for nuclear Ep-ICD accumulation, 25 events were observed. In contrast, no event was observed in 105 patients who did not show nuclear Ep-ICD immunopositivity and patients were alive for 60 months.

We evaluated the association between nuclear Ep-ICD accumulation, clinicopathological parameters and disease-free survival (Table 4). Significant association was observed between nuclear Ep-ICD expression in DCIS patients and disease-free survival (p < 0.001; Figure 3A). In contrast, all the 31 patients who did not show nuclear Ep-ICD positivity were alive and free of disease even after 5-years post-treatment. IDC patients also showed significant association between nuclear Ep-ICD expression and reduced disease-free survival (p < 0.001; Figure 3B). In contrast, all the 105 IDC patients with no nuclear Ep-ICD positivity were alive and free of disease as of 5-years following surgery. Among the IDC cases, Cox multivariate regression analysis showed nuclear Ep-ICD to be the most important prognostic marker for reduced disease-free survival (p = 0.011, HR = 80.18, 95% C.I. = 2.73–2352.2). Fifty of 75 nuclear Ep-ICD positive IDC patients did not have recurrence during this follow up period.

**Table 4:** **Kaplan-Meier survival analysis and multivariate Cox regression analysis for breast cancer patients**

***IDC Tumors***	Kaplan-Meier survival analysis unadjusted P-value	Multivariate Cox regression analysis adjusted P-value	Hazard’s Ratio (H.R.)	95% C.I.
**Nuclear Ep-ICD** ^**+**^	**<0.001**	**0.011**	**80.183**	**2.733–2352.2**
**Cytoplasmic Ep-ICD** ^**+**^	**0.048**	0.496	**----**	**----**
**Age**	0.796	0.787	**----**	**----**
**Tumor size**	0.556	0.516	**----**	**----**
**T-stage**	0.237	0.366	**----**	**----**
**Nodal status**	0.814	0.398	**----**	**----**
**Clinical Stage**	0.129	0.809	**----**	**----**
**Grade**	0.329	0.062	**----**	**----**
**ER status**	0.384	0.678	**----**	**----**
**PR status**	0.984	0.499	**----**	**----**

The p-value in boldface are statically significant.

## Discussion

Ever since the regulated intramembrane proteolysis of EpCAM was described as a novel mechanism of triggering oncogenic signalling by Maetzel *et al.* [31], investigation of Ep-ICD expression in human epithelial cancers for determination of its clinical relevance is in hot pursuit. Our earlier preliminary study reported frequent nuclear and cytoplasmic Ep-ICD expression in ten different epithelial cancers, including a small number of breast cancers [33]. This first report did not examine the correlation of nuclear Ep-ICD expression with clinical parameters or its prognostic utility in these cancers. The current study assessed the potential suitability of Ep-ICD as a marker in predicting clinical course and aggressiveness of breast cancer. Although expression of the full length EpCAM protein has been widely investigated in human malignancies, the expression and subcellular localization of its intracellular domain Ep-ICD has not been well characterized in clinical specimens. Our study demonstrated differences in expression of Ep-ICD and EpEx between normal and malignant breast tissues and their relationship with disease prognosis, providing valuable information as to their suitability as potential biological markers. Given the interest in the therapeutic potential of EpCAM targeted therapies in cancer management and the limited understanding of the role and expression pattern of Ep-ICD in breast cancer, our study helps to shed light on this widely-studied, yet not fully understood protein. Furthermore, our study is the first in-depth characterization of Ep-ICD expression in IDC of the breast.

Importantly, increased detection of nuclear Ep-ICD in breast carcinomas compared to normal tissues warrants exploration of its potential role in tumorigenesis. The increased regulated intramembrane proteolysis of EpCAM resulting in release of its cytoplasmic domain, Ep-ICD, and its subsequent translocation to the nucleus has been demonstrated to trigger oncogenic signalling in colon carcinoma [31]. In an earlier study, we reported that nuclear Ep-ICD accumulation predicted poor prognosis in thyroid carcinomas and was elevated in patients with anaplastic tumors [33]. Taken together with our present study, these reports underscore the biological significance of increased nuclear Ep-ICD in cancer. The discovery of the tumor-suppressive properties of EpCAM in some cancers has surprised many researchers, given its association with poor prognosis in many other cancers. Some studies have suggested the tumor microenvironment may be an important factor in dictating whether EpCAM will promote or inhibit tumor progression, particularly given its ability to mediate homophilic adhesive interactions between cells [10]. Furthermore, regulated intramembrane proteolysis of EpCAM and the associated oncogenic signalling by Ep-ICD may shed light on some of these observations as additional protein-protein interactions are uncovered [13, 36]. Recently, the endoplasmic reticulum aminopeptidase 2 (ERAP2), a proteolytic enzyme set in the endoplasmic reticulum (ER) has been shown to co-localize with EpCAM in the cytoplasm/ER where it plays a central role in the trimming of peptides for presentation by MHC class I molecules. This association between EpCAM and ERAP2 suggests a new mechanism of EpCAM processing and regulation of antigen presentation in breast cancer [37].

Our study revealed several important findings with potentially significant implications for the use of Ep-ICD as a biomarker. We observed high occurrence of recurrence, distal metastases, or death among IDC patients who were positive for nuclear Ep-ICD accumulation. In contrast, no recurrence distal metastases, or death were observed in nuclear Ep-ICD negative patients during the follow up period. Importantly, a great majority of patients with recurrence (37 of 42, 88.1%) had early stage breast carcinomas (AJCC pTNM Stage I and II) that would normally be considered lower-risk for future recurrence. Moreover, the fact that only nuclear Ep-ICD positive patients had recurrence and that no nuclear Ep-ICD negative patient suffered the same suggests a potential clinical application for this biomarker. These observations support the notion that nuclear Ep-ICD accumulation even in early stage breast tumors holds promise for predicting aggressive disease.

Indeed, the presence of nuclear Ep-ICD, irrespective of tumor stage or any other clinical variable predicted a high risk of disease recurrence. Multivariate Cox regression analyses identified nuclear Ep-ICD accumulation as the most significant factor for prediction of recurrence in IDC patients. These findings, of course, require further clinical validation in larger number of patients followed prospectively, but are nonetheless encouraging because it may provide a path to identify patients who may require more aggressive monitoring and/or treatment, particularly in patients early stage tumors who show nuclear Ep-ICD accumulation.

The *in vitro* studies on functional role of EpCAM in breast cancer cell lines demonstrated that transfection of EpCAM resulted in increased nuclear accumulation of β-catenin in MDA-MB-231^EpCAM^ and upregulated Wnt reporter assay activity in Hs578T^EpCAM^ cells suggesting activation of Wnt pathway [38]. Moreover, the interaction between membranous EpEx and the extracellular environment and nuclear Ep-ICD and intracellular signalling continues to reveal interesting associations. Martowicz *et al*. [39] and others recently reported that cancer cells of an epithelial but not mesenchymal phenotype require EpCAM as an invasion-promoting factor [40]. It is possible that nuclear Ep-ICD accumulation is an early indicator of tumor progression, as evidenced by its correlation with lower grade, but also, disease recurrence. Furthermore, the expression of nuclear Ep-ICD and membranous EpEx may have not only prognostic but also therapeutic implications to stratify patients who are likely to respond to EpCAM based immunotherapies. In this context, a recent study in 1365 breast cancers reported EpCAM expression varies significantly and is differentially associated with prognosis in the luminal B HER2 positive, basal like, and HER2 intrinsic subtypes of breast cancer [17–20]. However, a limitation of this study is the expression of Ep-ICD has not been analysed and only EpCAM expression was correlated with disease outcome. The prevalence of the full length EpCAM and Ep-ICD in a variety of human cancers has been recently reported using tissue microarrays suggesting loss of membranous EpEx is a common event in human epithelial cancers and the ratio of EpEx and Ep-ICD is dependent on the tumor [41]. However, this study does not address the clinical relevance of relative expression of EpEx and Ep-ICD in these cancers. Future studies evaluating the prognostic and predictive role of these variants in human cancers, especially in patients treated with Ep-CAM specific antibodies are warranted.

One limitation of our study is that while all the 25 IDC patients that had recurrence were nuclear Ep-ICD positive suggesting nuclear Ep-ICD positivity is a risk factor for aggressive disease in these patients, there were 50 of 75 nuclear Ep-ICD positive IDC patients who did not experience any recurrence during this follow up period. Hence there is a need to identify other protective factors in these patients that prevent the recurrence of disease. Another limitation is the very small number of ILC and IMC cases analyzed in this study. Future studies will be directed to search for additional factors which promote or protect against recurrence in this subgroup of nuclear Ep-ICD positive patients. Nevertheless, our findings are important in stratifying aggressive early stage breast cancer patients who will need rigorous follow-up for more effective disease management. At the same time the absence of nuclear Ep-ICD in early stage breast cancer patients also has the potential to help avoid over-treatment, sparing these patients the harmful side effects of aggressive therapies and reducing health care costs upon validation in future studies.

## Conclusions

In conclusion, nuclear Ep-ICD was detected in DCIS and IDC and found to be associated with recurrence in these patients. The recurrence of disease only in patients with nuclear Ep-ICD positive early stage tumors suggests that nuclear Ep-ICD accumulation holds the promise of identifying patients in need of more aggressive post-operative surveillance and/or treatment. Future studies investigating other factors that protect against recurrence in the subgroup of nuclear Ep-ICD positive patients are warranted to evaluate their prognostic significance. Clinical studies of Ep-ICD *vs.* membranous EpEx expression based selection of IDC patients who are likely to benefit from treatment with EpCAM-specific antibodies will unequivocally establish their utility for improving the outcome of EpCAM based molecular therapies.

## Authors’ information

Ranju Ralhan and Paul G Walfish are corresponding authors in this study.

## Abbreviations

CI: Confidence intervals
DCIS: Ductal carcinoma in situ
EGF: Epidermal growth factor
EpCAM: Epithelial cell adhesion molecule
Ep-ICD: Intracellular domain of EpCAM
EpEx: Extracellular domain of EpCAM
HR: Hazard ratio
IDC: Invasive ductal carcinoma
IHC: Immunohistochemistry
ILC: Invasive lobular carcinoma
IMC: Invasive mucinous carcinoma
LCIS: Lobular carcinoma in situ.
